# *LaPT2* Gene Encodes a Flavonoid Prenyltransferase in White Lupin

**DOI:** 10.3389/fpls.2021.673337

**Published:** 2021-06-11

**Authors:** Jinyue Liu, Yaying Xia, Wenbo Jiang, Guoan Shen, Yongzhen Pang

**Affiliations:** ^1^Institute of Animal Science, Chinese Academy of Agricultural Sciences, Beijing, China; ^2^Key Laboratory of Plant Resources and Beijing Botanical Garden, Institute of Botany, Chinese Academy of Sciences, Beijing, China; ^3^University of Chinese Academy of Sciences, Beijing, China; ^4^The Institute of Medicinal Plant Development, Beijing, China

**Keywords:** white lupin, flavonoids, prenyltransferase, LaPT2, kaempferol prenylation

## Abstract

Legume plants are rich in prenylated flavonoid compounds, which play an important role in plant defense and human health. In the present study, we identified a prenyltransferase (PT) gene, named *LaPT2*, in white lupin (*Lupinus albus*), which shows a high identity and close relationship with the other known PT genes involved in flavonoid prenylation in planta. The recombinant LaPT2 protein expressed in yeast cells exhibited a relatively strong activity toward several flavonols (e.g., kaempferol, quercetin, and myricetin) and a relatively weak activity toward flavanone (naringenin). In addition, the recombinant LaPT2 protein was also active toward several other types of flavonoids, including galangin, morin, 5-deoxyquercetin, 4'-O-methylkaempferol, taxifolin, and aromadendrin, with distinct enzymatic affinities. The *LaPT2* gene was preferentially expressed in the roots, which is consistent with the presence of prenylated flavonoid kaempferol in the roots. Moreover, we found that the expression level of *LaPT2* paralleled with those of *LaF3H1* and *LaFLS2* genes that were relatively higher in roots and lower in leaves, suggesting that they were essential for the accumulation of prenylated flavonoid kaempferol in roots. The deduced full-length LaPT2 protein and its signal peptide fused with a green fluorescent protein (GFP) are targeted to plastids in the *Arabidopsis thaliana* protoplast. Our study demonstrated that *LaPT2* from white lupin is responsible for the biosynthesis of prenylated flavonoids, in particular flavonols, which could be utilized as phytoalexin for plant defense and bioactive flavonoid compounds for human health.

## Introduction

Flavonoids, having a typical C6-C3-C6 skeleton structure, comprise one of the largest metabolite groups in the plant kingdom. Flavonoids are divided into a variety of classes; including flavones, flavonols, and isoflavonoids, depending on the levels of oxidation and the pattern of substitutions on their C ring (Middleton, 1998). These compounds possess very important physiological functions in plant growth and development and environment interaction (Dixon and Paiva, 1995).

The molecular basis for the formation of the different flavonoid skeletons has been well-documented, and the corresponding coding genes have been identified from various plant species, especially in model plants, such as *Arabidopsis thaliana*, *Zea mays*, and *Petunia hybrida* (Winkel-Shirley, 2001). Although the number of flavonoid skeletons is limited, a great number of diverse flavonoid compounds are subsequently synthesized by using a variety of modifications, including glycosylation, acylation, methylation, and prenylation (Dixon and Pasinetti, 2010). Prenylation is one of the specific modification forms of flavonoids, and prenylated flavonoids were widely presented in several plant families, including Leguminosae, Moraceae, Umbelliferae, and Euphorbiaceae (Tahara and Ibrahim, 1995; Barron and Ibrahim, 1996; Boland and Donnelly, 1998; Botta et al., 2005, 2009).

Prenylated flavonoids display important physiological functions in response to environmental stress (Sohn et al., 2004). In addition, prenylated flavonoids also possess considerable health-promoting properties for human health (De Naeyer et al., 2004; Ahmed-Belkacem et al., 2005; Han et al., 2006). In particular, prenylated flavonols, such as the methylated 8-prenylkaempferol (also called sophoflavescenol), showed cytotoxicity against the different cancer cell lines as well as antidiabetic and anti-Alzheimer activities (Jung et al., 2011; Boozari et al., 2019).

Prenylation of flavonoid compounds was catalyzed by a prenyltransferase (PT) from the plant microsomal fraction (Yamamoto et al., 2000; Zhao et al., 2003). Several genes encoding flavonoid PTs have been isolated and characterized from various plant species, in particular legume plants, including *SfN8DT*, *SfG6DT*, and *SfiLDT* from *Sophora flavescens* (Sasaki et al., 2008, 2011; Chen et al., 2013), *GmG4DT* and *GmG2DT* from *Glycine max* (Akashi et al., 2009; Yoneyama et al., 2016; Sukumaran et al., 2018), *LjG6DT* from *Lotus japonicus* (Liu et al., 2018), and *GuA6DT* and *GuILDT* from *Glycyrrhiza uralensis* (Li et al., 2014, 2018). Their recombinant proteins expressed in yeast microsomal fractions could utilize the different flavonoid aglycones as an acceptor, and dimethylallyl pyrophosphate (DMAPP) as a donor to introduce prenyl moiety at C6, C8, or C3' positions of specific flavonoids. However, in all these cases, none of the PTs could catalyze the prenylation of flavonols, a large group of bioactive flavonoids in planta.

White lupin (*Lupinus albus*) is a widely cultivated legume plant with an excellent and a high content of proteins in seeds, which was first domesticated in the Mediterranean region and was planted in many countries in the world (Lucas et al., 2015). White lupin accumulates various prenylated flavonoid compounds, and both the structure and physiological property of these flavonoids have been well-characterized (Tahara et al., 1984; Gagnon et al., 1992; Katagiri et al., 2000; Bednarek et al., 2001). However, reports on PT genes in the flavonoid pathway are still rare in white lupin. In a previous study, we identified an *LaPT1* gene from white lupin, and the encoding PT protein could prenylate genistein at C3' position to produce isowighteone (Shen et al., 2012), but additional genes encoding PT that could prenylate various flavonoids remain unknown in white lupin.

In order to identify additional PT genes in this prenylflavonoid-rich plant species, we further searched the white lupin transcriptome database and isolated another PT gene of *LaPT2*. *In vitro* enzymatic assays showed that the recombinant LaPT2 protein could use several different flavonoid compounds as acceptors, and DMAPP as a donor to generate prenylated flavonoids. In addition, we found that the presence of prenylkaempferol in roots is consistent with a relatively high expression level of the *LaPT2* gene in the roots of white lupin. The discovery of the *LaPT2* gene could provide a valuable reference for the in-depth investigation of the functional diversification and evolution of PT in planta, and metabolic engineering of bioactive prenylated flavonoids as medicines for human health.

## Materials and Methods

### Plant Materials and Growth Conditions

White lupin seeds (*L. albus*) were obtained from the United States Department of Agriculture Soybean Germplasm Collection. The seeds were germinated and grown in vermiculite under the controlled condition of 16/8 h of light/dark cycles at 25°C. The leaves and roots of 30-day-old were harvested separately and stored at −80°C until further analysis.

### Chemicals

Flavonoid standards, such as genistein, 2'-hydroxygenistein, daidzein, apigenin, kaempferol, 3'-methylkaempferol, quercetin, myricetin, 3-hydroxyflavone, naringenin, liquiritigenin, and isoliquiritigenin, were purchased from Shanghai Tongtian Biotechnology Company Ltd. (Shanghai, China), and catechin, 5-deoxyquercetin, galangin, morin, and kaempferol-3-*O*-glucoside were obtained from Chemfaces Biochemical Co. Ltd. (Wuhan, China), and 8-prenylated kaempferol was obtained from Yuanye Biotechnology Company Ltd. (Shanghai, China). DMAPP, farnesyl diphosphate (FPP), and geranyl diphosphate (GPP) were purchased from Sigma-Aldrich (St. Louis, MO, United States).

### Phylogenetic Analysis of Plant PTs

The protein sequences of PTs involved in the natural product biosynthesis were used for the sequence alignment and construction of the phylogenetic tree. The multi-sequence alignment was performed by using Clustal X2 (Larkin et al., 2007). Then, the molecular phylogenetic tree based on the Maximum Likelihood Method was reconstructed by using the MEGA6 software with a bootstrap value of 1,000 (Tamura et al., 2013).

### *In vitro* PT Activity Assays

The construction of a vector pDR196GW-LaPT2, yeast transformation in the W303A1 strain, and microsomal protein extraction were the same as previously reported (Liu et al., 2018). In brief, the total microsomal protein was quantified by using the Bradford (1976) assay and immediately used for PT activity assays. The enzymatic reaction contained 1 mM of dithiothreitol, 25 mM of MOPS (pH 7.0), 10 mM of Mg^2+^, 100 μM of flavonoid substrates, and 20 μg of total microsomal protein. The reaction mixture of 200 μl was incubated at 30°C for 30 min and then terminated by the addition of 200 μl of methanol. The apparent *Km* values for flavonoid substrates were determined with various concentrations of flavonoid substrates (ranging from 5 to 400 μM), 10 μg of the total microsomal protein with a final volume of 50 μl. The apparent *Km* values for DMAPP (ranging from 50 to 400 μM) were determined by using 400 μM kaempferol. The mixtures were incubated at 30°C for 30 min, and the products were quantified by using the standard curve of corresponding flavonoid substrates through a peak area of the UV spectrum on high-performance liquid chromatography (HPLC). All reactions were performed in triplicates. The apparent *Km* values were calculated by using the Eadie–Hofstee Plot. The conversion rates (%) were calculated as follows: peak area for a product/peak area for a substrate in control reactions.

### Subcellular Localization of LaPT2 Protein

The open reading frame of LaPT2 lacking the stop codon, the 87-amino-acid transit peptide fragment, and the fragment lacking the 87-amino-acid were, respectively, subcloned into the pJIT163-*green fluorescent protein* (GFP) vector, resulting in the LaPT2-GFP, LaPT2-TP_1-87_-GFP, and LaPT2_88-403_-GFP fusion gene under the control of a double 35S CaMV promoter. These three plasmids were further confirmed by sequencing and transforming into the *Arabidopsis* protoplasts *via* the polyethylene glycol (PEG)-mediated transformation as previously described (Yoo et al., 2007). After 16 h of incubation at 25°C, the GFP fluorescence was observed under a confocal laser scanning microscope (Leica TCS SP5, Germany).

### Gene Expression Analysis by Quantitative Real-Time PCR

**Figure 1:**
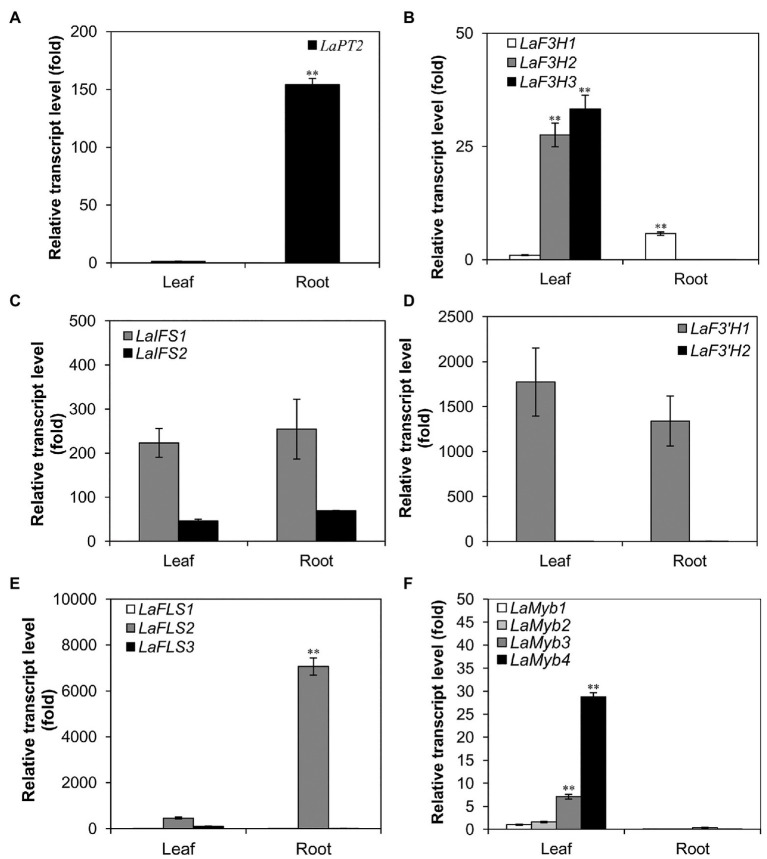
Expression profile of *LaPT2* in leaves and roots of white lupin using quantitative real-time PCR (qRT-PCR) analysis. **(A–F)** Relative transcript levels of *LaPT2* **(A)**, three *LaF3H* **(B)**, two *LaIFS* **(C)**, two *LaF3'H* **(D)**, three *LaFLS* **(E)**, and four *LaMYB* **(F)** in leaves and roots of white lupin. In **(A)** the expression level of *LaPT2* in leaves was set to 1; in **(B,C)** the expression level of *LaF3H1* in leaves was set to 1. In **(D)** the expression level of *LaF3'H2* in leaves was set to 1. In **(E)** the expression level of *LaFLS1* in leaves was set to 1. In **(F)** the expression level of *LaMyb1* in leaves was set to 1. Data are presented as mean ± SD, Student’s *t*-test (*n* = 3, ^∗∗^*p* < 0.01, comparison between leaves and roots samples for each gene) with three technical duplicates.

For the analysis of gene expression in different organs, leaves and roots were separately harvested from 30-day-old white lupin plants grown in vermiculite. The collected samples were immediately frozen in liquid nitrogen and grounded into a fine powder. Total RNAs were extracted by using RNAiso Plus (code No. 9108, Takara, Japan) according to the instructions of the manufacturer. Total RNAs were treated with DNase I to digest genomic DNA and were then used as a template to synthesize the first-strand complementary DNA (cDNA) by using reverse transcriptase moloney murine leukemia virus (M-MLV; RNase H-, code No. 2641A, Takara, Japan). The cDNA was used as a template for the quantitative real-time PCR (qRT-PCR) analysis with the SYBR Green PCR kit (Kangwei Biotech, Beijing, China). The qRT-PCR analyses were performed by using gene-specific primers (Supplementary Table 2). The transcript levels of each gene were determined by relative quantification using the 2^-ΔΔCt^ method and normalized by using the *actin* gene as a reference. The data were indicated as an average of biological and technical triplicates. Further, the lowest expression level of a gene in each panel (Figure 1) was set at 1, and the data were indicated as an average of three replicates.

### Analysis of Flavonoid Metabolites

For the analysis of flavonoid compounds, leaf and root materials (about 1 g fresh weight) were harvested and lyophilized; 20 mg of the sample (dry weight) was extracted with 1 ml of 80% methanol. An analysis of 20 μl extracts was performed on HPLC of Agilent 1260 Infinity II (Santa Clara, CA, United States) with a linear eluting gradient (5–70% solvent B over 30 min, 70–100% solvent B in 30–35 min) with solvent A (0.1% formic acid in water) and solvent B (0.1% formic acid in acetonitrile) at the flow rate of 1 ml/min, with Eclipse XDB-C18 reverse-phase column (4.6 mm × 150 mm, 5-Micron, Agilent, Santa Clara, CA, United States) or ultra-performance liquid chromatography (UPLC) in Agilent 1290 Infinity II (Santa Clara, CA, United States) with a linear eluting gradient (5–40% solvent B over 7 min, 40–100% solvent B in 7–8.5 min, 100% solvent B in 8.5–10 min, and 100–5% solvent B in 10–10.1 min) with the same solvent A and solvent B at the flow rate of 0.4 ml/min with Eclipse Plus C18 reverse-phase column (2.1 mm × 100 mm, 1.8-Micron, Agilent, Santa Clara, CA, United States). A photodiode array detector (Agilent, Santa Clara, CA, United States) was used for the detection of UV-visible absorption from 190 to 600 nm. The quantification of flavonoids was through the peak area of the UV spectrum on HPLC. The products were also analyzed on a Xevo triple quadrupole mass spectrometer (TQ-MS, Waters, MA, United States) in a negative-ion mode, and the MS detection conditions were as follows: capillary voltage, 2.50 kV; cone voltage, 70 V; desolvation gas flow, 650 L/h; cone gas flow, 50 L/h; collision gas flow, 0.12 ml/min; collision energy, 30 eV; desolvation temperature, 350°C; source temperature, 150°C; and scan range, 100–1, 000 mass-to-charge ratio (*m*/*z*).

### Overexpression of *LaPT2* in *A. thaliana*

To test the *in vivo* function of LaPT2, it was subcloned into the binary vector pCAMBIA2300-35S-OCS between the sites of *Kpn* I and *Sal* I. *Agrobacterium tumefaciens* strain GV3101 clones containing pCAMBIA2300-35S-OCS-LaPT2 were used for transformation into *Arabidopsis* by using the floral dip method (Clough and Bent, 1998). T2 generation seeds were germinated on plates with MS containing 50 mg/ml kanamycin, and the resistant seedlings were transferred to soil to obtain homozygous T3 generation seeds.

For the flavonoid analysis, transgenic *Arabidopsis* was grown at 22°C with 16/8 h light and dark cycles. The 21-day-old seedlings were collected, freeze-dried, and used for the flavonoid extraction by using the 80% method and detected on HPLC by using the abovementioned method.

For feeding assays, both wild-type and transgenic *Arabidopsis* plants (2-week-old seedlings) were grown on plates with or without 0.1 mM kaempferol, and flavonoids were extracted with 80% aqueous acetone and further processed as described by Sasaki et al. (2008). The final methanol extracts from the wild-type and transgenic plants were analyzed on HPLC/MS by using the abovementioned method.

## Results

### Identification of a Candidate Flavonoid PT Gene *LaPT2* in White Lupin

In a previous study, we identified a PT gene *LaPT1*, which is responsible for the prenylation of isoflavone genistein and 2'-OH genistein at C3' position in white lupin (Shen et al., 2012). To further identify additional candidate genes for the biosynthesis of diverse prenylated flavonoids in white lupin, we performed a blast search with *LaPT1* as a query against the white lupin transcriptome database containing 65,097 contigs (Secco et al., 2014). Two EST sequences (LAGI02_4454 and LAGI02_33083) with the highest identity (about 45%) were obtained, and they were 1,892 and 1, 460 bp in length, encoding deduced proteins of 407 and 402 amino acids in length, respectively.

**Figure 2:**
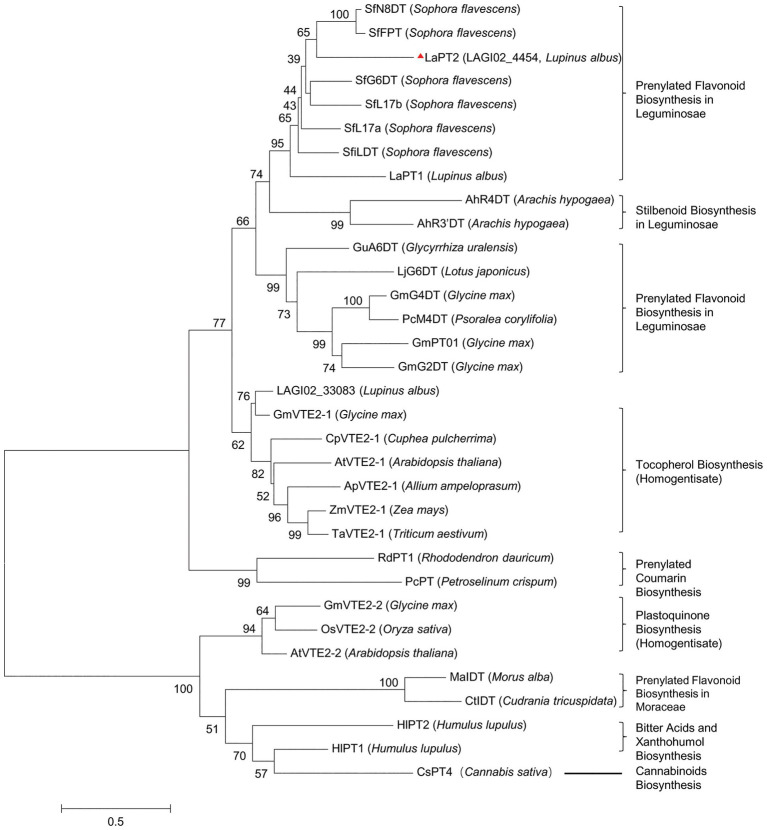
Phylogenetic analysis of the deduced prenyltransferases (PTs) from white lupin with other closely related PTs in plant species. The multi-sequence alignment was performed by using Clustal X2. Then, the molecular phylogenetic tree based on the maximum likelihood method was reconstructed by using the MEGA6 software with a bootstrap value of 1,000. The Genbank accession number are as follows: AtVTE2-1, AY089963; ApVTE2-1, DQ231057; CpVTE2-1, DQ231058; MaIDT, KM262659; CtIDT, KM262660; AtVTE2-2, DQ231060; HIPT-1, AB543053; HIPT-2, KM222442; SfFPT, KC513505; SfN8DT-1, AB325579; SfG6DT, AB604224; SfiLDT, AB604223; LaPT1, JN228254; GmG2DT, LC140930; GmPT01, KRH76147.1; GmG4DT, AB434690; GuA6DT, KJ123716; LjG6DT, KX228696; SfL17a, AB371287; SfL17b, AB370329; LaPT2, MH298812; AhR4DT, AQM74172.1; AhR3’DT, AQM74173.1; PcM4DT, AYV64464.1; PcPT, W0SKS3.1; RdPT1, BBD96134.1; GmVTE2-2, ABB70128.1; OsVTE2-2, BAC83059.1; OsVTE2-2, BAC83059.1; ZmVTE2-1, ABB70122.1; TaVTE2-1, ABB70123.1; GmVTE2-1, ABB70126.1; and CsPT4, DAC76710.1.

A phylogenetic tree with other plant PTs involved in flavonoids, vitamin E, and plastoquinone biosynthesis showed that the deduced protein encoded by LAGI02_4454 was grouped within the same clade containing several flavonoid PTs from Leguminosae, whereas the deduced protein encoded by LAGI02_33083 was clustered with PTs involved in tocopherol biosynthesis (Figure 2). Meanwhile, the deduced protein encoded by LAGI02_33083 showed a higher identity with AtVTE2-1 compared with LaPT1. Both sequence identity and phylogenetic analysis highly suggested that LAGI02_4454 was most likely to be involved in the flavonoid biosynthesis, whereas LAGI02_33083 was involved in the tocopherol biosynthesis.

Therefore, we isolated the open reading frame of LAGI02_4454 (GenBank accession No. MH298812) using RT-PCR and designated it as *LaPT2*. The deduced LaPT2 protein showed 59, 56, 49, and 48% sequence identity with SfG8DT, SfG6DT, GuG6DT, and LaPT1, respectively, in the amino acid level. The LaPT2 protein contained two conserved PT motifs, NQxxDxxxD and KD(I/L)xDx(E/D), which were shared by other flavonoid PTs (Supplementary Figure 1). LaPT2 was predicted to possess seven putative transmembrane domains (Supplementary Figure 1), which was similar to the other characterized plant flavonoid PTs with seven to nine transmembrane domains (Supplementary Figure 1).

### *In vitro* Function of the Recombinant LaPT2 Protein

In order to test the *in vitro* function of the recombinant LaPT2 protein, its open reading frame was cloned into the yeast expression vector pDR196GW. The plasmid pDR196GW-LaPT2 was transformed into the yeast strain W303A1. The extracted microsomal proteins containing LaPT2 were initially incubated with Mg^2+^ as a cofactor and eight representative flavonoid aglycones as the potential substrates (genistein, 2'-hydroxygenistein, daidzein, naringenin, liquiritigenin, isoliquiritigenin, apigenin, and kaempferol).

**Figure 3:**
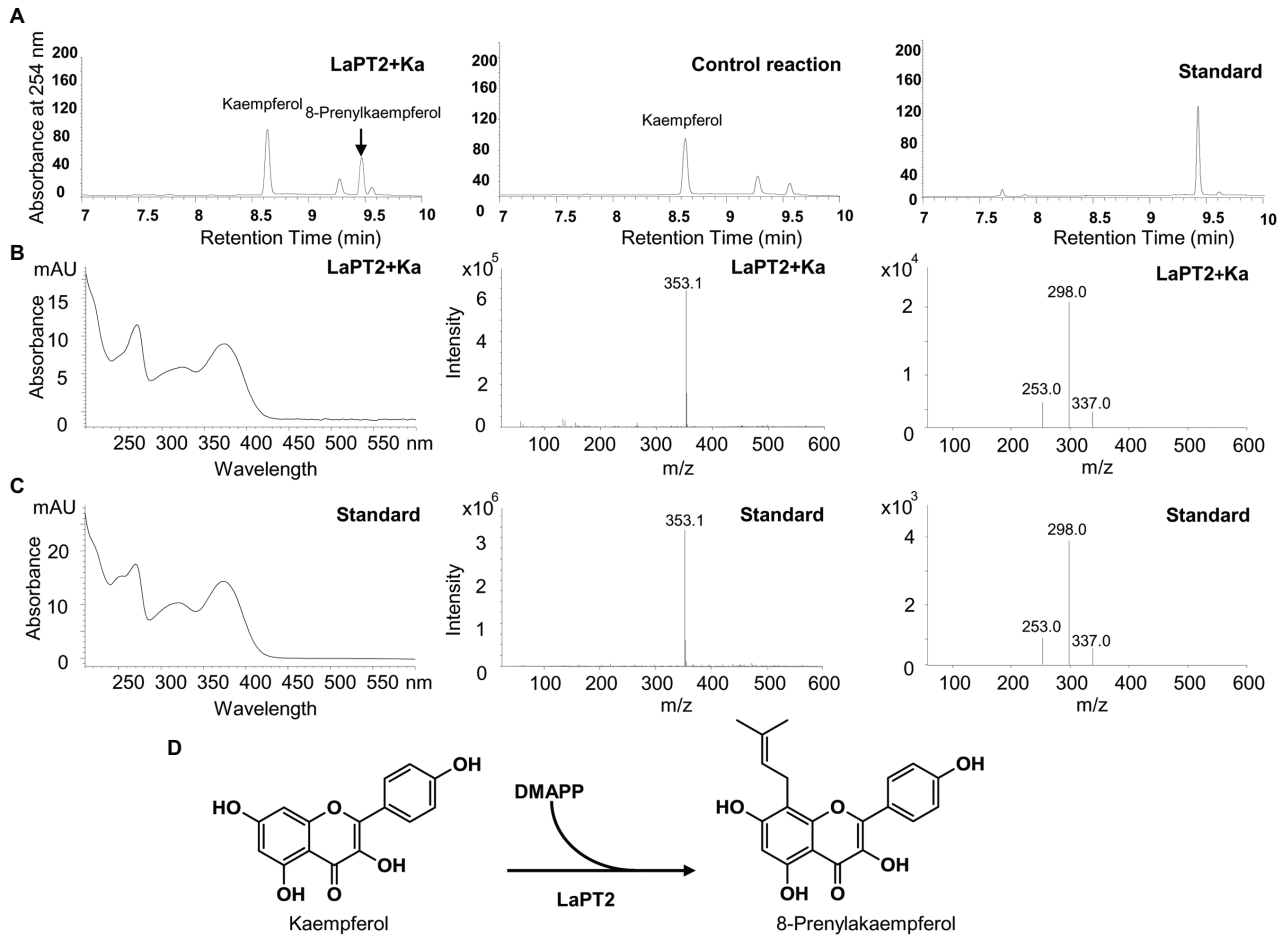
Identification of the enzymatic product of the recombinant LaPT2 protein by high-performance liquid chromatography (HPLC), ultra-performance liquid chromatography (UPLC)/mass spectrometer (UPLC/MS), and UPLC/MS/MS analyses. **(A)** Representative HPLC chromatographs of the enzymatic reactions containing kaempferol (Ka), dimethylallyl pyrophosphate (DMAPP), and a microsomal fraction of yeast expressing LaPT2 (left), control reaction (middle), and the 8-prenylkaempferol standard (right). **(B,C)** UV chromatograph (left), mass spectrum (middle), and MS/MS spectrum (right) of the enzymatic product **(B)** and the standard **(C)**, respectively. **(D)** Chemical structures of kaempferol substrate and product catalyzed by the recombinant LaPT2 protein.

It was revealed that the recombinant LaPT2 protein could not accept genistein, 2'-hydroxygenistein, daidzein, liquiritigenin, isoliquiritigenin, or apigenin as the substrate, but showed an activity toward kaempferol and naringenin (Figure 3; Supplementary Figure 2). New products were detected by using HPLC in the different reactions with kaempferol and naringenin as a substrate (Figure 3A, left; Supplementary Figure 2A), in comparison with the control reactions (Figure 3A, middle; Supplementary Figure 2B). The new enzymatic products with naringenin as a substrate were confirmed to be a prenylated flavonoid naringenin with monoisotopic *m/z* of 339.4 by both UPLC-MS (Supplementary Figure 2C) and UPLC/MS/MS (Supplementary Figure 2D). In particular, the new enzymatic products with kaempferol as a substrate were further confirmed to be 8-prenylkaempferol (Figure 3B) as compared with the UV chromatograph (Figures 3B,C, left), MS (Figures 3B,C, middle), and MS/MS (Figures 3B,C, right) of the available authentic standard (Figure 3C), indicating that LaPT2 was able to prenylate kaempferol at the C8 position (Figure 3D).

### Enzymatic Properties of the Recombinant LaPT2 Toward Other Flavonoid Substrates

**Figure 4:**
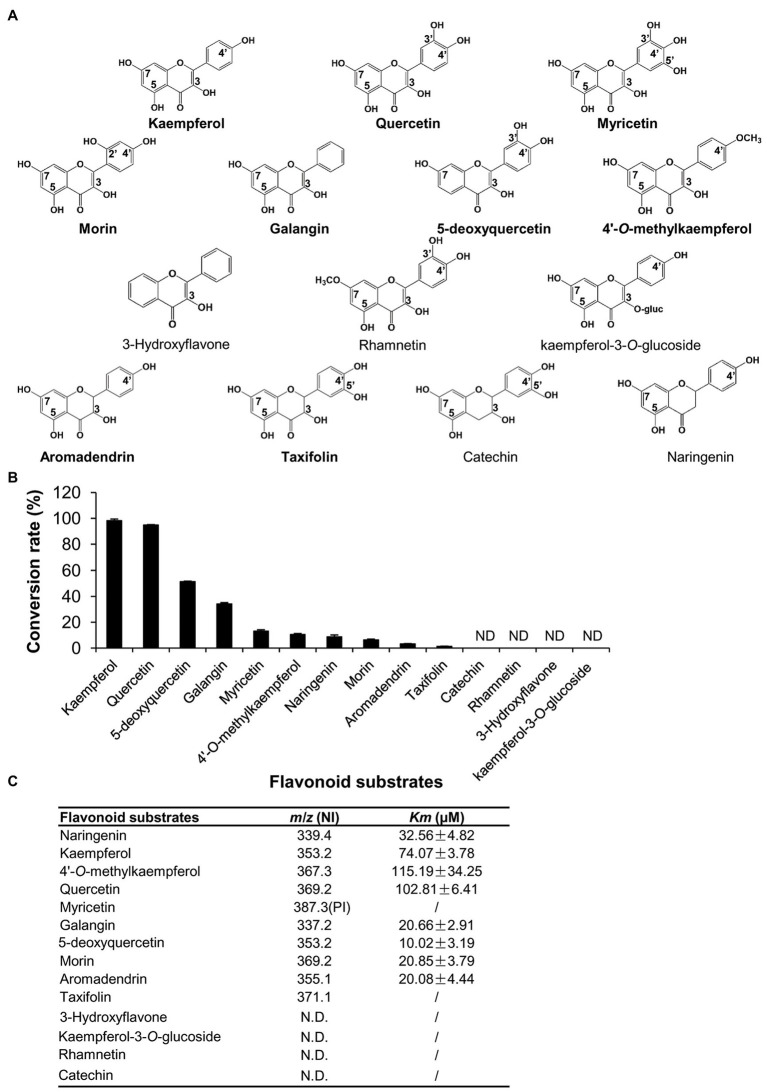
Comparison of the enzymatic efficiency of the recombinant LaPT2 protein toward different flavonoid substrates. **(A)** Chemical structures of the representative flavonoid substrates tested in the present study. **(B)** The conversion rate of the recombinant LaPT2 protein toward different flavonoid substrates. **(C)** Apparent *Km* values of the recombinant LaPT2 protein toward different flavonoid substrates. *m/z* is for mass-to-charge ratio, NI is for negative-ion mode, and PI is for positive-ion mode. The enzymatic assays were performed with technical triplicates in **(B,C)**.

Because LaPT2 could prenylate representative flavonol of kaempferol, it could possibly also use other flavonols or additional flavonoids as substrates. Therefore, other 12 available flavonoid compounds, including quercetin, myricetin, morin, galangin, 5-deoxyquercetin, 3-hydroxyflavone, 4'-*O*-methylkaempferol, rhamnetin, kaempferol-3-*O*-glucoside, aromadendrin, taxifolin, and catechin, were further tested as potential substrates (Figure 4A).

It was revealed that the recombinant LaPT2 protein could use quercetin, myricetin, and morin as substrates, and they all have hydroxyl groups at C3, C5, C7, and C4' positions (Figure 4A; Supplementary Figures 3A–C). In addition, the recombinant LaPT2 protein could convert galangin (hydroxy group at C3, C5, and C7 positions), 5-deoxyquercetin (hydroxy group at C3, C7, C3', and C4' positions), and 4'-*O*-methylkaempferol (hydroxy group at C3, C5, and C7 positions) to the corresponding prenylated products (Figure 4A; Supplementary Figures 3D–F). However, 3-hydroxy flavone (without hydroxyl groups at either C5 and C7 or C4' positions) and rhamnetin (a methoxy group is substituted at C7 position of quercetin) could not be used as a substrate (Figure 4A), implying that the OH group at the C7 position is critical with the OH groups at C3 and C5 being important but not essential.

As mentioned above, LaPT2 is not active toward apigenin that lacks OH group at C3 position, but it was active toward naringenin that also lacks OH group at C3 position. However, kaempferol-3-*O*-glucoside (an *O*-glycosyl group at C3 position) could not be prenylated like kaempferol (Figure 4); therefore, these data together suggested that the OH group at C3 position is critical at least for flavonols. In addition, we also found that two dihydro flavonols, aromadendrin, and taxifolin could be prenylated (Supplementary Figures 3G,H), but another structurally similar flavanol compound, catechin, could not be accepted as a substrate (Figure 4A).

The relative conversion rates of LaPT2 toward flavonoid compounds were in the order of kaempferol (98.25%) > quercetin (94.85%) > 5-deoxyquercetin (51.46%) > galangin (34.10%) > myricetin (13.16%) > 4'-*O*-methylkaempferol (10.59%) > naringenin (8.76%) > morin (6.41%) > aromadendrin (3.29%) > taxifolin (1.27%; Figure 4B), implying kaempferol and quercetin, without any additional glucosylation or methylation modification, are the preferred substrates for LaPT2. Furthermore, the C2–C3 double bond is important for LaPT2 to accept the flavonols better than the other flavonoid skeletons.

In addition, the apparent *Km* values of kaempferol, 4'-*O*-methylkaempferol, and quercetin were revealed to be 74.07 ± 3.78, 115.19 ± 34.25, and 102.81 ± 6.41 μM, respectively, and they were higher than those of galangin, 5-deoxyquercetin, morin, and aromadendrin, with the apparent *Km* values of 20.66 ± 2.91, 10.02 ± 3.19, 20.85 ± 3.79, and 20.08 ± 4.44 μM (Figure 4C). This result implied that kaempferol, 4'-*O*-methylkaempferol, and quercetin showed less affinity toward LaPT2 than galangin, 5-deoxyquercetin, morin, or aromadendrin as far as their apparent *Km* values were concerned.

In addition to DMAPP, we also detected FPP and GPP as the prenyl donor with kaempferol as a substrate. It was revealed that neither FPP nor GPP could be accepted as a prenyl donor (Supplementary Figures 4A,B), but only DMAPP with an apparent *Km* value of 66.11 ± 7.54 μM. Taken together, it is clear that LaPT2 is a flavonoid PT with DMAPP as a preferred prenyl donor and flavonols as the preferred substrates.

### Subcellular Localization of LaPT2

The LaPT2 protein contains a putative transit peptide of 87 amino acids at its N-terminus for targeting the chloroplast as predicted by ChloroP 1.1. To verify its subcellular localization, the full-length LaPT2 protein fused with GFP (LaPT2-GFP), its 87-amino-acid transit peptide fused with GFP (LaPT2_1-87_-GFP), and the truncated protein fused with GFP (LaPT2_88-403_-GFP, lacking the transit peptide) were, respectively, introduced into *Arabidopsis* protoplasts for the subcellular localization analysis.

**Figure 5:**
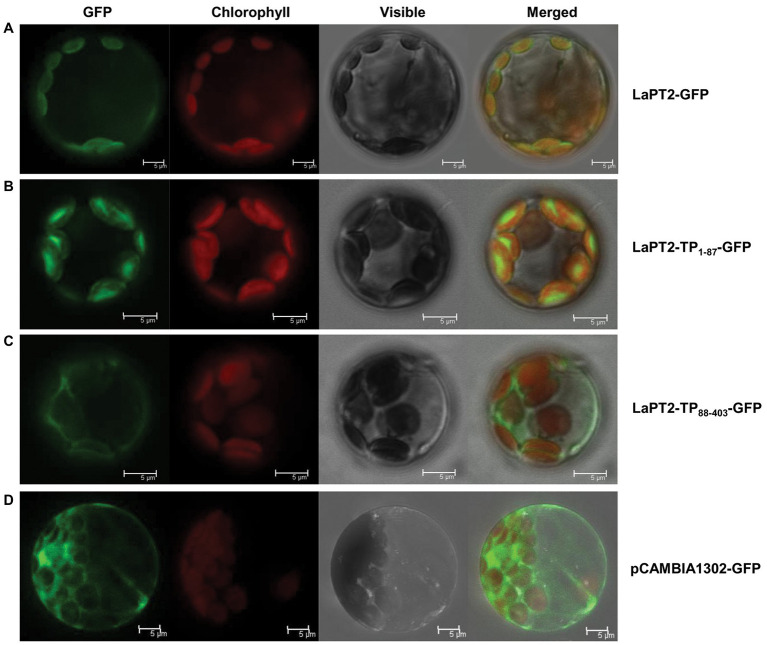
Subcellular localization of the LaPT2 protein in *Arabidopsis* protoplasts. **(A)** Fluorescence signals from the protoplast transiently expressing the LaPT2-green fluorescent protein (GFP) construct were observed under laser confocal scanning microscopy. Bars = 5 μm. **(B)** Fluorescence signals from the protoplast transiently expressing the LaPT2_1-87_-GFP construct, which encoded only the transit peptide sequence of LaPT2. Bars = 5 μm. **(C)** Fluorescence signals from the protoplast transiently expressing the LaPT2_88-403_-GFP construct, which lacked the transit peptide sequence of LaPT2. Bars = 5 μm. **(D)** Fluorescence signals from the protoplast transiently expressing pCAMBIA1302-GFP as control. Bars = 5 μm.

Green fluorescence of the native LaPT2-GFP fusion protein was detected in protoplasts, which was overlapped with the red autofluorescence of the chloroplasts (Figure 5A). In addition, a strong green fluorescence of LaPT2_1-87_-GFP was clearly observed in the chloroplasts, which was the same as for LaPT2-GFP (Figures 5A,B). In contrast, green fluorescence of the LaPT2_88-403_-GFP fusion protein without the signal peptide was not found in the chloroplasts (Figure 5C), which was similar to that of the free GFP control (Figure 5D). These results clearly indicated that LaPT2 is localized to the chloroplasts, and the 87-amino-acid signal peptide is essential and in itself alone could be targeted to the correct/valid subcellular organelle.

### Expression Pattern of the *LaPT2* Gene

To further elucidate the expression pattern of *LaPT2* in white lupin, its expression levels in leaves and roots were determined by using qRT-PCR, along with several other genes involved in the flavonol and isoflavone biosynthetic pathway. It was shown that the *LaPT2* gene was preferentially expressed in roots than in leaves (Figure 1A).

Meanwhile, the expression levels of several other pathway genes were also determined, including three *flavanone 3-hydroxylase* (*F3H*; *LaF3H1*, *2*, and *3*), two *2-hydroxyisoflavanone synthases* (*IFS*; *LaIFS1* and *2*), two *F3'H* (*LaF3'H1* and *2*), three *flavonol synthases* (*FLS*; *LaF3H1*, *2*, and *3*), and four *Myb* transcription factor genes (*LaMyb1*, *2*, *3*, and *4* that showed a relatively high similarity to the *MYBs* involved in the flavonoid pathway from the other plant species; Figures 1B–F). In the present study, their relative expression levels in leaves and roots were compared with the gene-specific primers (Figures 1B–F; Supplementary Table 2). Some of them were expressed with a relatively high level, including *LaFLS2* in roots (Figure 1E), *La F3'H1* and *LaIFS1* in both leaves and roots (Figures 1C,D). Among all these genes, only *LaF3H1* and *LaFLS2* showed the same expression pattern as *LaPT2*, with relatively higher expression levels in roots than in leaves, although the expression level of *LaF3H1* is lower than that of *LaFLS2* (Figures 1B,E). This result implied that *LaPT2* likely co-express with *LaF3H1* and *LaFLS2* for the production of prenylated flavonols in the roots of white lupin.

### Flavonoid Profiles in White Lupin

**Figure 6:**
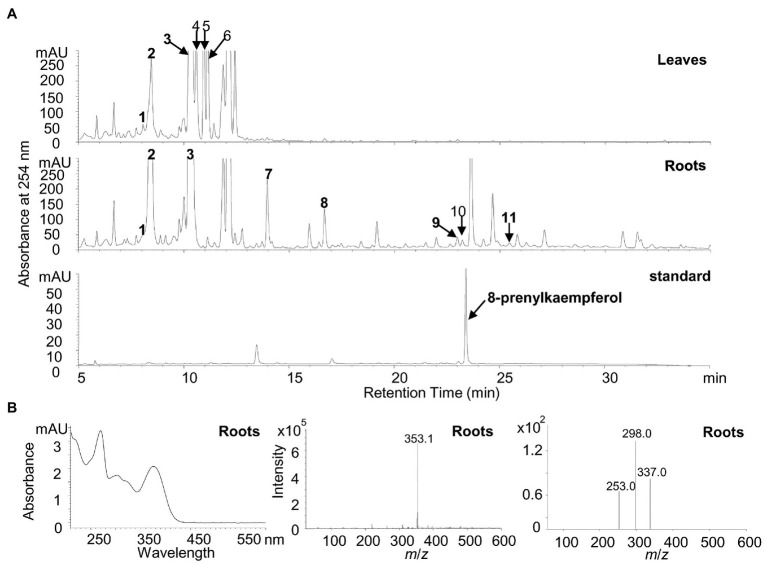
Identification of putative flavonoid compounds in leaves and roots of white lupin using HPLC, UPLC/MS, and UPLC/MS/MS. **(A)** Representative HPLC chromatographs of the methanolic extracts from white lupin leaves (upper panel), roots (middle panel), and the 8-prenylkaempferol standard (lower panel). Numbers indicated identified flavonoids as shown in Supplementary Table 1. **(B)** UV chromatograph (left), mass spectrum (middle), and tandem mass spectrum (right) of 8-prenylkaempferol identified in roots.

To detect the potential presence of prenylated flavonols in white lupin, the leaves and roots of a 30-day-old white lupin were harvested to profile flavonoid compounds by using the HPLC, the UPLC-MS, and the UPLC-MS-MS analysis. In total, 11 peaks corresponding to the 12 compounds (Figures 6A,B; Supplementary Table 1) were identified, supported by UV chromatographs (Supplementary Figure 5), mass spectrum (Supplementary Figure 6), and tandem mass spectrum (Supplementary Figure 7), and referred with a previous report (D’Agostina et al., 2008). Among them, seven isoflavonoids and five flavonol conjugates were detected in the leaves and/or roots (Supplementary Table 1). In particular, isorhamnetin 3-*O*-galactoside (peak 6 in Figure 6A), kaempferol 3-*O*-glucoside (peak 4 in Figure 6A), and 2'-hydroxygenistein 4'-*O*-glucoside (peak 2 in Figure 6A) were relatively abundant in the leaves whereas 2′-hydroxygenistein 4'-*O*-glucoside (peak 2 in Figure 6B) and genistein 7-*O*-glucoside (peak 3 in Figure 6B) were relatively abundant in the roots (Supplementary Table 1). However, isorhamnetin 3-*O*-galactoside and kaempferol 3-*O*-glucoside were absent in the roots although they were relatively abundant in the leaves (Figure 6A; Supplementary Table 1).

In particular, it was found that a peak at the same retention time for the 8-prenylkaempferol standard was identified only in the roots (Figure 6B) but not in the leaves, although it was buried with another putative compound (m/z 351.1) that showed different UV chromatographs, MS spectrums, and MS/MS spectrums (Supplementary Figures 5–7 for peak 10). The peak in the roots for 8-prenylkaempferol was very small, which indicates a trace amount, but the ion fragment at the same retention times was the same as the authentic standard (m/z 353.1; Supplementary Figure 8). Taken together, the presence of 8-prenylkaempferol in the roots was confirmed by comparison with the authentic standard using the same UV chromatograph, MS, and MS/MS spectrum (Figure 6B; Supplementary Figure 8).

### Overexpression of *LaPT2* in *A. thaliana*

To verify the *in vivo* function of *LaPT2*, it was overexpressed in *A. thaliana* that is rich in flavonol kaempferol in the leaves. Three independent lines that showed high expression levels of *LaPT2*, which were confirmed by using qRT-PCR (Supplementary Figure 9A), and the flavonoid profiles in the seedlings were analyzed on HPLC. No significant difference or distinct new peaks were detected in the transgenic lines in comparison with the wild type and the 8-prenylkaempferol standard (Supplementary Figure 9B). In addition, we further fed the seedlings with kaempferol on agar plates and detected the flavonoid profiles using the UPLC/MS. However, only the level of kaempferol 3-*O*-glucoside-7-*O*-rhamnoside (K3G7R) was increased in both transgenic and the wild-type lines (Supplementary Figures 9C,D), no additional new peak/fragment was detected by searching a targeted ion fragment of 353 for prenylkaempferol.

## Discussion

Several prenylated flavonoid compounds have been isolated from the different plant species, especially in Leguminosae. Some legume plant species accumulate prenylated flavonoids, such as gluceollins in soybean (Schmidt et al., 1992) and wighteone in *L. japonicus* (Liu et al., 2018), under environmental stress, whereas some plants constitutively produce prenylated flavonoids under the normal growth condition, as in white lupin (Tahara et al., 1984; Gagnon et al., 1992; Katagiri et al., 2000). In the present study, we identified another PT gene *LaPT2* in white lupin and found that it encodes a putative flavonoid PT as revealed by both sequence and phylogenetic analyses (Figure 2; Supplementary Figure 1). The deduced LaPT2 protein was localized in the chloroplast (Figure 5), which is the same as for the other characterized flavonoid PTs (Sasaki et al., 2008; Akashi et al., 2009; Shen et al., 2012). Further study on subcellular localization confirmed that the transit peptide of LaPT2 carries the localization signal, and the deletion of the N-terminus signal peptide sequence leads to an incorrect localization (Figure 5), which is the same as for the other PT (Sasaki et al., 2008; Akashi et al., 2009; Shen et al., 2012; Liu et al., 2018), and all these studies demonstrated that the presence of a signal peptide is essential for the correct localization of PT proteins.

### Flavonoid Accumulation and the Expression Level of Related Pathway Genes

**Figure 7:**
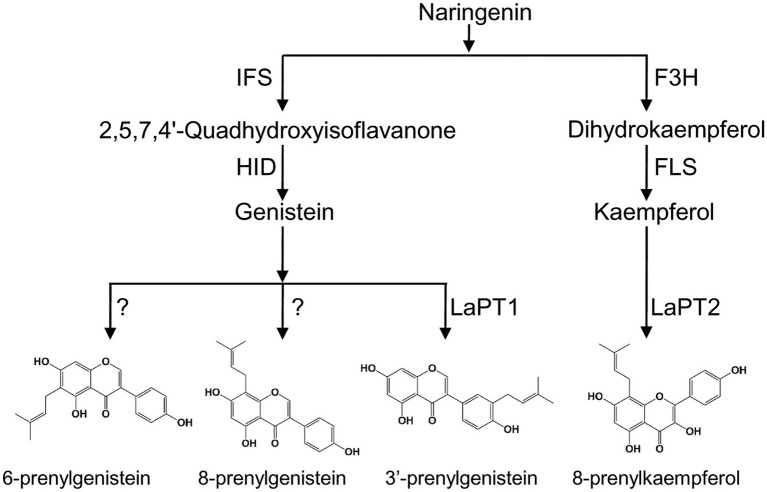
Proposed biosynthetic pathway of prenylated flavonoids in white lupin. F3H, flavanone 3-hydroxylase; FLS, flavonol synthase; IFS, 2-hydroxyisoflavanone synthase; HID, 2-hydroxyisoflavanone dehydratase; and PT, prenyltransferase.

Legume plants produce a large number of flavonoid compounds, which are the result of complex biosynthesis and regulation. Our present study showed that white lupin accumulates both isoflavonoid and flavonol conjugates in leaves and roots (Figure 6). We found that the isoflavonoid and flavonol accumulation pattern was associated with the expression pattern of the corresponding key pathway genes (Figures 1, 6, 7). The relatively higher expression levels of *LaIFS1* in both leaves and roots may explain the abundance of glucosides of genistein and 2'-hydroxygenistein in both leaves and roots (Figures 1C, 6A,B; Supplementary Table 1). Although *LaF3*'*H1* was highly expressed in both leaves and roots, the quercetin conjugate isorhamnetin 3-*O*-galactoside was accumulated at a high level in leaves but not in roots (Figures 1D, 6A,B; Supplementary Table 1), indicating that the other genes rather than *LaF3*'*H1* might be a key gene for the biosynthesis of isorhamnetin 3-*O*-galactoside and other quercetin conjugates in white lupin. Although four *Myb* genes were detected, their relative expression levels were not high in comparison with other structural genes, indicating that they may not be the key genes for the flavonoid accumulation in white lupin.

In addition, the prenylated compounds (luteone, wighteone, and prenylkaempferol) in white lupin were detected and showed relatively high levels in roots than in leaves, in the current and in the previous study (D’Agostina et al., 2008), which is associated with a relatively high level of expression of *LaPT2* genes in this study and the *LaPT1* gene in the previous study (Shen et al., 2012). It was clear that roots might be a desirable tissue for further discovery and identification of additional flavonoid PT genes in white lupin. Meanwhile, except 8-prenylkaempferol, the prenylated products of other flavonoids identified in *in vitro* enzymatic assays for LaPT2 were not discovered in a white lupin plant, and the inconsistence behavior of *in vitro* and *in vivo* activity was also observed for the PTs identified in other plant species like *S. flavescens* (Yazaki et al., 2009; Chen et al., 2013). In another aspect, *LaFLS2* coexpressed with *LaPT2* in the roots; therefore, it might be more ideal to produce a prenylated flavonoid by combining structural genes (e.g., *LaFLS2* and *LaPT2*) *in vitro* for commercial purposes.

On the other hand, leaves of the wild type *A. thaliana* accumulate mainly kaempferol conjugates, indicating the presence of kaempferol, but no prenylated kaempferol was detected when *LaPT2* was overexpressed. Even when kaempferol was fed into the transgenic *A. thaliana*, no additional prenylated kaempferol was produced (Supplementary Figure 9). This result was consistent with the fact that kaempferol glucosides could not be prenylated in the *in vitro* enzymatic assays owing to preferential modification of kaempferol as glucosides or rhamnosides in the leaves of *A. thaliana*, whereas *LaPT2* is expected to act in roots. In addition, the feeding of kaempferol in *A. thaliana* has led to a more metabolic flux to K3G7R (Supplementary Figure 9), which may be explained by the competition between the prenylation and glycosylation of flavonoids in planta. Recent studies on *Artocarpus heterophyllus* showed that the substrate specificity of flavonoid PTs AhPT1 may change with a different cofactor, i.e., Mn^2+^ instead of Mg^2+^. As Mn^2+^ is an essential cofactor for photosynthetic activity, it might be accumulated in the chloroplast exposed to light and to which LaPT2 might be also targeted (Yang et al., 2020). In addition to these, the other factors may also affect the *in vivo* enzymatic activity of LaPT2 in ectopic plant species; therefore, the *in vivo* function and properties of LaPT2 protein require further investigation in the near future.

### LaPT2 Preferred Flavonol Aglycones as Substrates

The LaPT2 protein showed the closest relationship with SfG6DT and LaPT1 in the phylogenetic tree, and white lupin accumulates a significant amount of prenylated isoflavonoids, but LaPT2 exhibited no enzymatic activity toward isoflavones, which is different from SfG6DT and LaPT1. The results indicated that LaPT2 was able to catalyze the prenylation of flavonol kaempferol, and flavanone naringenin, which is the same as for SfN8DT (Sasaki et al., 2008). However, LaPT2 displayed a higher conversion rate toward kaempferol than naringenin, which was different from SfN8DT that was more active toward naringenin than kaempferol (Sasaki et al., 2008). With the presence of prenylated flavonoid kaempferol in white lupin (even though it was in trace amount and buried with another putative compound) and the high conversion rate of LaPT2 toward kaempferol, it is reasonable to speculate that kaempferol serves as the natural substrate for LaPT2 in white lupin.

Most flavonoid PTs from legume plants displayed strictly the substrate specificity (Akashi et al., 2009; Sasaki et al., 2011; Shen et al., 2012; Liu et al., 2018). Among all flavonoid-specific PTs described previously, only GuA6DT from *G. uralensis* and *Sophora flavescens* flavonoid prenyltransferase (SfFPT) from *S. flavescens* displayed relatively broad substrate specificity toward flavonoid compounds with a similar structure (Chen et al., 2013; Li et al., 2014). In our study, the recombinant LaPT2 protein showed a catalytic activity toward more flavonol-type than the other types of flavonoids (Figure 4). The fact that LaPT2 did not catalyze 3-hydroxy flavone indicated that one and/or more hydroxyl groups at C4', C5, and C7 positions of the flavonol skeleton were critical for the recognition of prenylation substrates. Moreover, the double bond between C2 and C3 is important for LaPT2 to accept flavonols as preferred substrates than the other flavonoid skeletons.

Many flavonol aglycones are modified through the addition of hydroxyl and/or methoxy groups on the B-ring based on the skeletal structure of kaempferol. Although LaPT2 is active toward various flavonol compounds, the maximum conversion efficiency was observed for kaempferol under the same condition (Figure 4). The prenylation reaction catalyzed by the PT represented a Friedel-Crafts alkylation of the flavonoid skeleton in the biosynthesis of prenylflavonoids (Chen et al., 2013; Li et al., 2014). The hydroxyl groups at the C7/C5 position of A-ring and C3'/C4' position of B-ring would increase prenylation efficiency of SfFPT while the methoxy group on the abovementioned positions would decrease efficiency (Chen et al., 2013). It was similar for LaPT2 that the presence of methoxy group did decrease the prenylation efficiency owing to the decrease in the conversion rate from kaempferol to 4'-*O*-methoylkaempferol and from quercetin to rhamnetin (no activity for rhamnetin; Figure 4) whereas the presence of hydroxyl groups at C7/C5 increased prenylation efficiency owing to the increase in the conversion rate from 5-deoxyquercein to quercetin (Figure 4). However, the hydroxyl group at C-3'/C4' reduces the prenylation efficiency for LaPT2 as the conversion rate decreased from kaempferol, quercetin to myricetin, from galangin to morin, and from aromadendrin to taxifolin (Figure 4B), which is different from SfFPT (Chen et al., 2013).

In this study, we found that LaPT2 could not accept kaempferol-3-*O*-glucoside as a substrate, which was similar to SfFTP that could not prenylate glycosylated flavanone (Chen et al., 2013). Therefore, it was hypothesized that prenylation precedes glycosylation in the production of complicated flavonoid compounds, for example, the prenylation reaction precedes the glycosylation step in epimedoside, a biosynthesis in *Epimedium diphyllum* (Yamamoto et al., 1997). Meanwhile, glucosylation may stabilize the accumulation of transit prenylated flavonoids in a plant as the detection of trace amounts of prenylkaempferol in the roots of white lupin (Figure 6).

White lupin produces different types of prenylisoflavones (Tahara et al., 1984; D’Agostina et al., 2008), but only one PT gene *LaPT1* was identified to be able to prenylate isoflavonoid (2'OH) genistein at C3' position and LaPT2 prefers flavonols as a substrate but not isoflavonoids (Figure 7). We speculate that more flavonoid PT genes were present in the white lupin genome, besides *LaPT1* and *LaPT2*. On the basis of the BLAST search, a few more candidate PT EST sequences with low identity to *LaPT1* were also yielded, and their functions were still under further investigation for the full elucidation of the prenylation mechanism of flavonoids in legume plants.

## Data Availability Statement

The datasets presented in this study can be found in online repositories. The names of the repository/repositories and accession number(s) can be found in the article/Supplementary Material.

## Author Contributions

JL, GS, and YP conceived the project and designed the experiments. JL, YX, and WJ performed the experiments and analyzed the data. JL, YX, and GS prepared the manuscript. YP revised the manuscript and supervised the project. All authors contributed to the article and approved the submitted version.

### Conflict of Interest

The authors declare that the research was conducted in the absence of any commercial or financial relationships that could be construed as a potential conflict of interest.
