# Supplementary material for: LaPT2 Gene Encodes a Flavonoid Prenyltransferase in White Lupin
Source: Front Plant Sci. 2021 Jun 11;12:673337. doi: 10.3389/fpls.2021.673337 (PMC8226212; doi:10.3389/fpls.2021.673337)
Supplement: Supplementary file 1 [file Data_Sheet_1.PDF]

Supporting information

Supplemental Table 1. Identification of flavonoids in white lupin by UPLC/MS and MS/MS analyses.

peak	compound	t_R(min)	MS/MS	Leaves (mg/g)	Roots (mg/g)
**1**	**2'-hydroxygenistein 7-*O*-glucoside**	**8.259**	**447.0;285.0**	**0.22±0.04**	**0.24±0.02**
**2**	**2'-hydroxygenistein 4'-*O*-glucoside**	**8.441**	**447.0;285.0**	**2.03±0.41**	**5.19±0.80**
**3**	**genistein 7-*O*-glucoside**	**10.254**	**431.0;268.0**	**1.62±0.17**	**3.48±0.13**
4	kaempferol 3-*O*-glucoside	10.579	447.0;284.0	2.04±0.72	-
5	kaempferol 3-*O*-galactoside	10.935	447.0;284.0	+[a]	-
	isorhamnetin 3-*O*-glucoside		477.1;314.0	+[a]	-
6	isorhamnetin 3-*O*-galactoside	11.116	477.1;314.0	4.38+0.55	-
**7**	**2'-hydroxygenistein**	**13.943**	**285.0;217.0;175.0**	**0.24±0.11**	**0.62±0.07**
**8**	**genistein**	**16.66**	**269.0;224.0;133.0**	**0.06±0.03**	**0.51±0.13**
**9**	**luteone**	**22.957**	**353.1;285.1;219.0;133.0**	**0.03±0.01**	**0.11±0.02**
10	8-prenylkaempferol	23.199	353.1;337.0;298.0;253.0;164.0	-	+[a]
	putative compound		351.0;283.0;199.0;151.0	-	+[a]
**11**	**wighteone**	**25.424**	**337.1;281.0;201.0**	**+[b]**	**0.11±0.01**

Note: +[a]: double compounds and could not be separated for quantification; +[b]: the amount is too low and could not be quantified; -: not detected. The informaiton on isoflavonoid compounds were highlighted in bold.

Supplemental Table 2. Primer name and sequences used in the present study.

Gene name	mRNA accession number	Primer name	Primer sequence (from 5' to 3')
**Protein Expression in yeast**			
LaPT2	LAGI02_4454	LaPT2Spe1F	GGACTAGTATGGGTTTTGTGCTTGCAGCTAC
LaPT2	LAGI02_4454	LaPT2Xho1R	GTGCTCGAGTCATCTAAATAAAGGTATGAGG
**Subcellular Localization**			
LaPT2	LAGI02_4454	LaPT2SubLSal1F	ACGCGTCGACATGGGTTTTGTGCTTGCAGCTAC
LaPT2	LAGI02_4454	LaPT2SubLBamH1R	CGCGGATCCTCTAAATAAAGGTATGAGG
LaPT2	LAGI02_4454	LaPT2SLBamH1R$_{87}$	CGCGGATCCTTCATTTGATTTTCCAGAGGCT
LaPT2	LAGI02_4454	LaPT2SLSal1F$_{\triangle 1\text{-}87}$	ACGCGTCGACATGTATGAAACCCAAGATCTTGATC
**qRT-PCR Analysis**			
LaPT2	LAGI02_4454	LaPT2 qF	GCAGCTACTTCTTTTCCCAAAG
LaPT2	LAGI02_4454	LaPT2 qR	ATTTCTCTTGTCTCCCCCTTTG
Actin		LaactinRTF2	TGGACGACCTCGTCATACTG
Actin		LaactinRTR2	AAGCATCCTTCTGACCCATC
LaIFS2	rLAGI02_1754	LaIFS2 qF	CATCCACCACTTCCTGTGGTT
LaIFS2	rLAGI02_1754	LaIFS2 qR	ACACTAAGTCCTGGCCTCTCT
LaIFS1	LAGI02_33262	LaIFS1 qF	AGCCGTGGCAACAGACTATG
LaIFS1	LAGI02_33262	LaIFS1 qR	TCCCCTCAGATCAACTGGGT
LaMyb4	LA02_38569	LaMYB4 qF	ACAAGGGAAGTGGTGGTTCAA
LaMyb4	LA02_38569	LaMYB4 qR	CCAAGGCCTCCTCAACACTAC
LaMyb3	LA02_38159	LaMYB3 qF	CGTTGTCGGAGACACGGAAT
LaMyb3	LA02_38159	LaMYB3 qR	TAACTCAACGCCCCACCAAC
LaMyb1	LA02_44741	LaMYB1 qF	GTGAGGACAGTGGTGCAGTT

LaMyb1	LA02_44741	LaMYB1 qR	CTTTCCAAAGCCAAGTGAGCA
LaMyb2	LA02_2514	LaMYB2 qF	TGCCTCAAAGGAATGCAACTG
LaMyb2	LA02_2514	LaMYB2 qR	GCTCCTCCTTAAGGGTCACAT
LaF3H1	LAGI02_43731	LaF3H1 qF	AAGCAGTGCAGGATTGGAGAG
LaF3H1	LAGI02_43731	LaF3H1 qR	TGGTTCCTCCAACATAGGCTTC
LaF3H2	La02_41747_71057	LaF3H2 qF	AGAAGATTGTGGAGGCGTGT
LaF3H2	La02_41747_71057	LaF3H2 qR	TCCACCCTTCTGGCTTGTCT
LaF3H3	LAGI02_54973	LaF3H3 qF	AGCCGTCTATCCATAGCCAC
LaF3H3	LAGI02_54973	LaF3H3 qR	TTGGCCATGTCGAAGTTTTGC
LaFLS1	LAGI02_45697	LaFLS1 qF	GGCATCACAACGGTTCATGG
LaFLS1	LAGI02_45697	LaFLS1 qR	ACACTTTGTCTACCACGCCA
LaFLS2	rLAGI02_2306	LaFLS2 qF	AAGGGTACAAACTGTGGCATC
LaFLS2	rLAGI02_2306	LaFLS2 qR	TGATGCCTGGTTGTTCTGTCT
LaFLS3	rLAGI02_904	LaFLS3 qF	AAGCTGCAGGTGGAGATAGC
LaFLS3	rLAGI02_904	LaFLS3 qR	TTGCACGTCATTGGGGACAA
LaF3’H1	rLAGI02_34328	LaF3’H1 qF	GTTGGCCGGAGCTCTCAATA
LaF3’H1	rLAGI02_34328	LaF3’H1 qR	ATTGGGGTCACGTGCTATGG
LaF3’H2	LAGI02_35407	LaF3’H2 qF	AATTTCTCTAGCCGGCCACC
LaF3’H2	LAGI02_35407	LaF3’H2 qR	CAGTGAGTCTGCCTCCATCC
**Over-expression in *Arabidopsis thaliana***			
LaPT2	LAGI02_4454	LaPT2KpnIF	CGGGGTACCATGGGTTTTGTGCTTGCAGCTAC
LaPT2	LAGI02_4454	LaPT2SalIR	ACGCGTCGACTCATCTAAATAAAGGTATGAGG

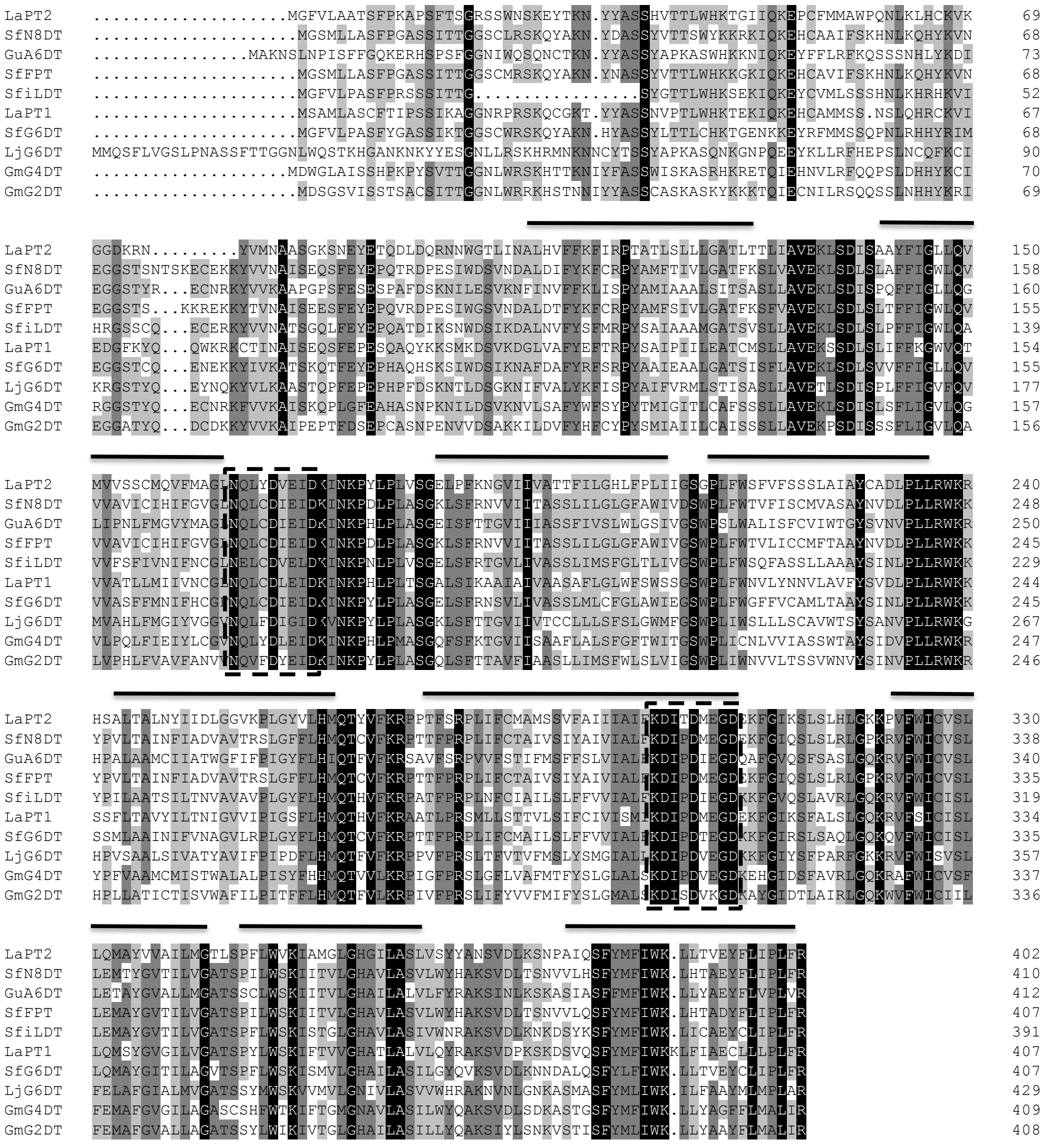

Figure S1. Sequence analyses of the deduced prenyltransferases from *Lupinus albus* with other closely related flavonoid-specific prenyltransferases in Leguminosae.

Multiple sequence alignment of the two deduced prenyltransferases from *Lupinus albus* with other related flavonoid-specific prenyltransferases. Identical amino acids are shown in black background and similar amino acids are in gray background. The two conserved NQxxDxxID and KDI/LxDxE/DGD motifs are boxed, and the transmembrane domains were indicated in solid lines.

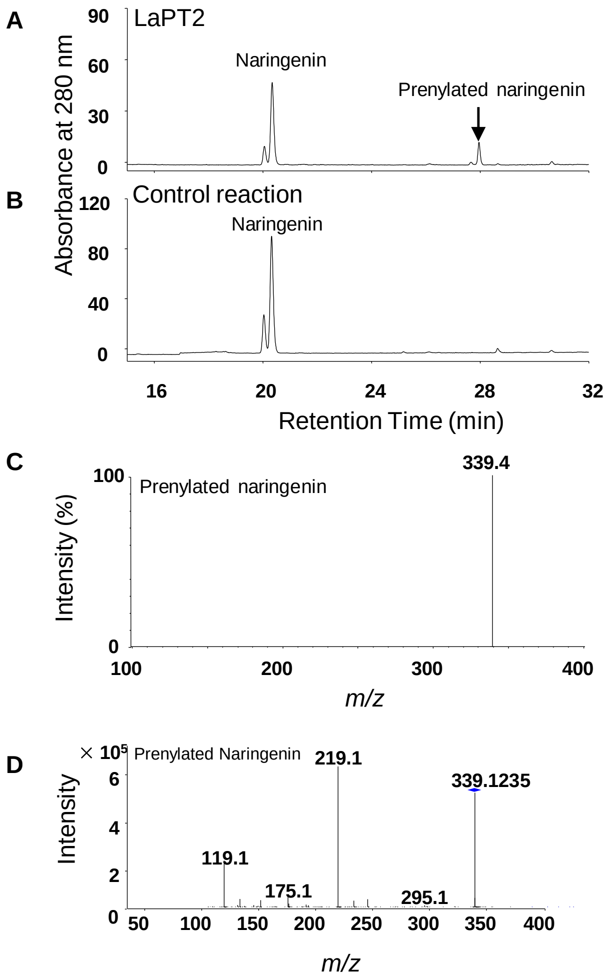

Figure S2. Identification of the enzymatic product of recombinant LaPT2 protein by HPLC, UPLC/MS and UPLC/MS/MS analyses with naringenin as substrate. (A-D) Representative HPLC chromatographs of the enzymatic reaction containing naringenin, DMAPP and microsomal fraction of yeast expressing LaPT2 (A), control reaction (B), mass spectrum of the enzymatic product of recombinant LaPT2 protein determined by UPLC/MS (C), and MS/MS spectrum of the enzymatic product (D), respectively.

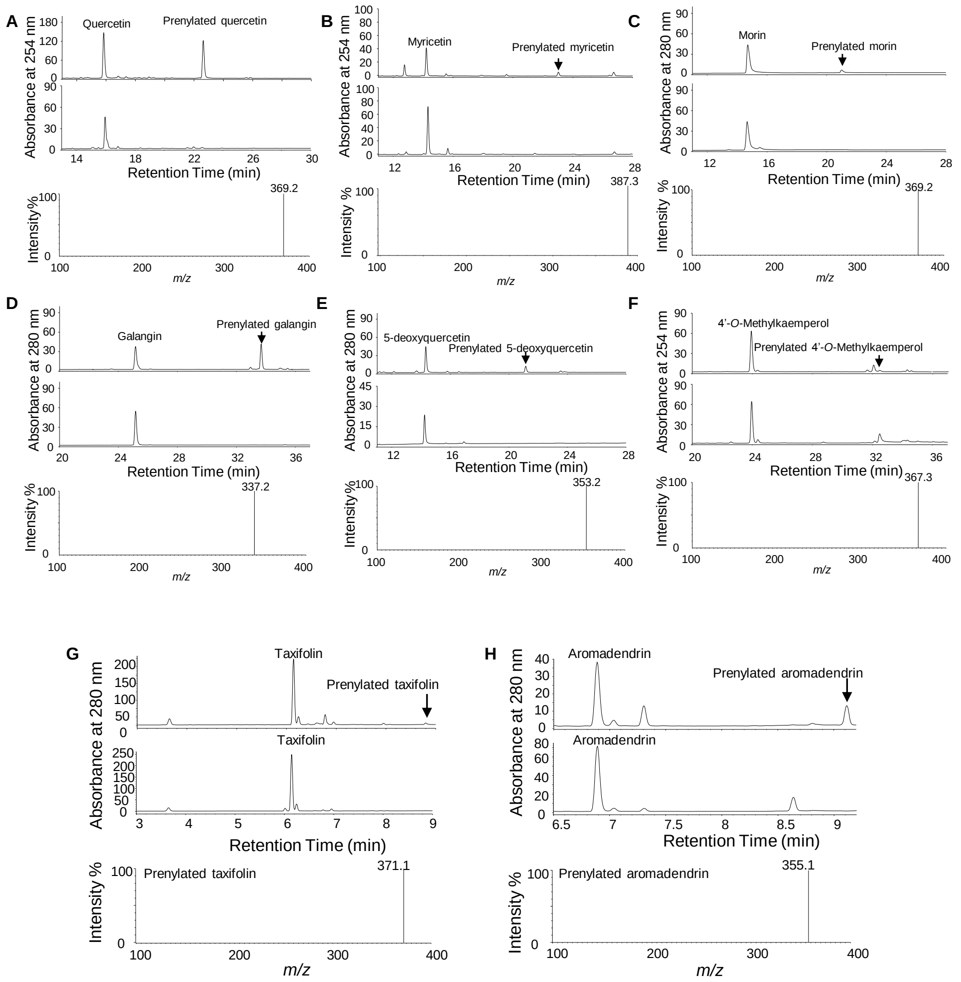

Figure S3. Identification of the enzymatic product of the recombinant LaPT2 protein by HPLC and UPLC/MS analyses.

(A-H) Representative HPLC chromatographs of the enzymatic reactions containing quercetin (A), myricetin (B), morin (C), galangin (D), 5-deoxyquercetin (E), 4’-O-methylkaempferol (F), taxifolin (G), and aromadendrin (H), DMAPP and microsomal fraction of yeast expressing LaPT2 (upper panel), control reaction (middle panel), tandem mass spectrum of the enzymatic product of recombinant LaPT2 protein determined by UPLC/MS (lower panel), respectively.

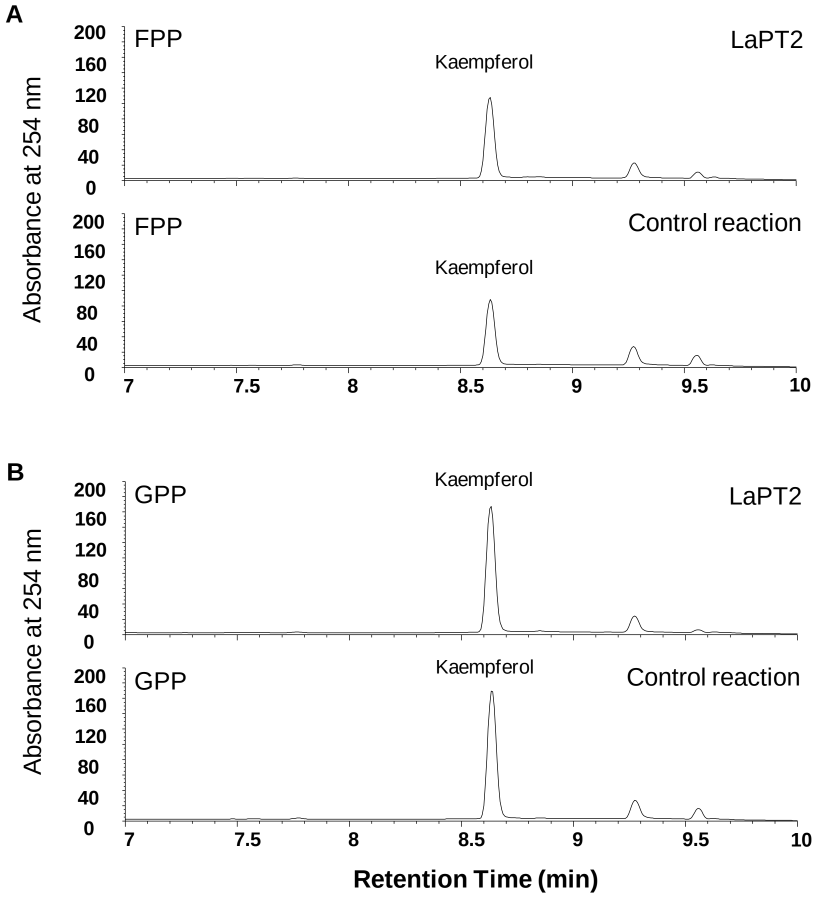

Figure S4. Analyses of the enzymatic product of the recombinant LaPT2 protein by HPLC with FPP and GPP as prenyl donors.

(A-B) Representative HPLC chromatographs of the enzymatic product containing kaempferol as substrate, FPP (A), GPP (B) as different prenyl donor, with microsomal fraction of yeast expressing LaPT2 (upper panel) or control reaction (lower panel), respectively.

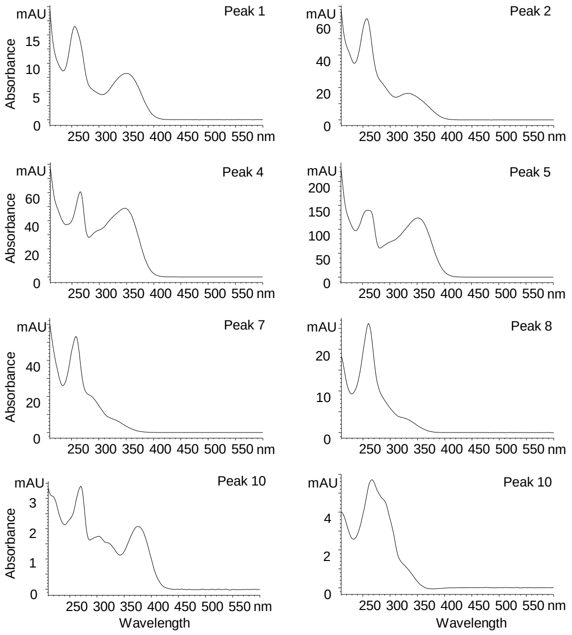

Figure S5. Identification of the major flavonoid compounds in white lupin by UV chromatograph analysis.

UV chromatographs of flavonoid compounds from white lupin for peaks 1-11 as shown in Table S1.

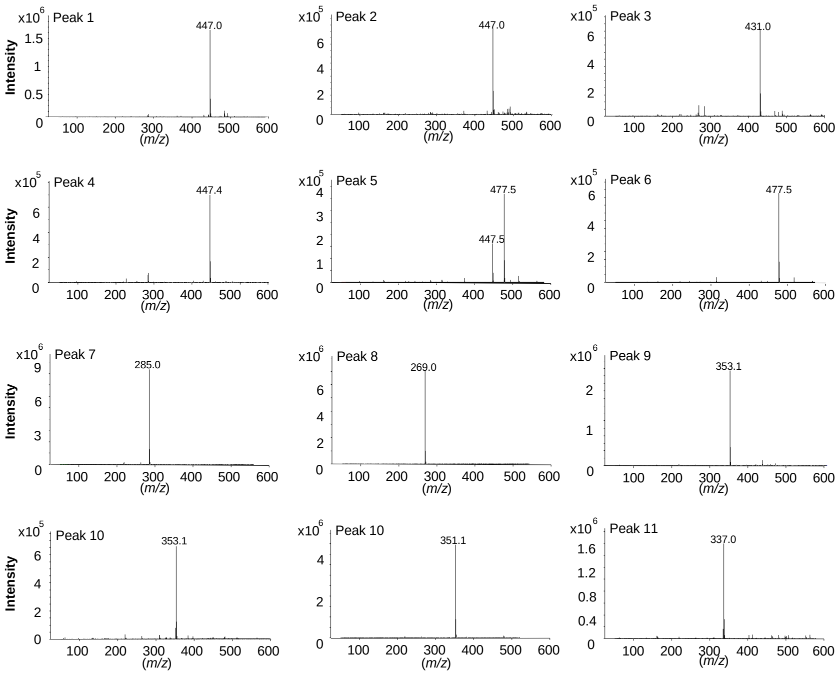

Figure S6. Identification of the major flavonoid compounds in white lupin by mass spectrum.

Mass spectrum of flavonoid compounds from white lupin for peaks 1-11 as shown in Table S1.

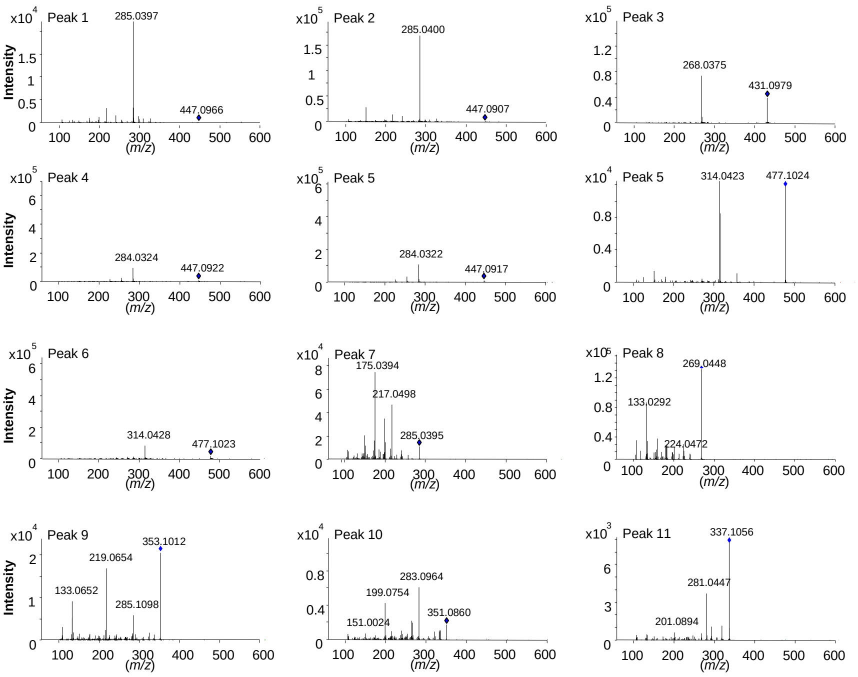

Figure S7. Identification of the major flavonoid compounds in white lupin by tandam mass spectrum.

MS/MS analyses of flavonoid compounds from white lupin for peaks 1-11 as shown in Table S1.

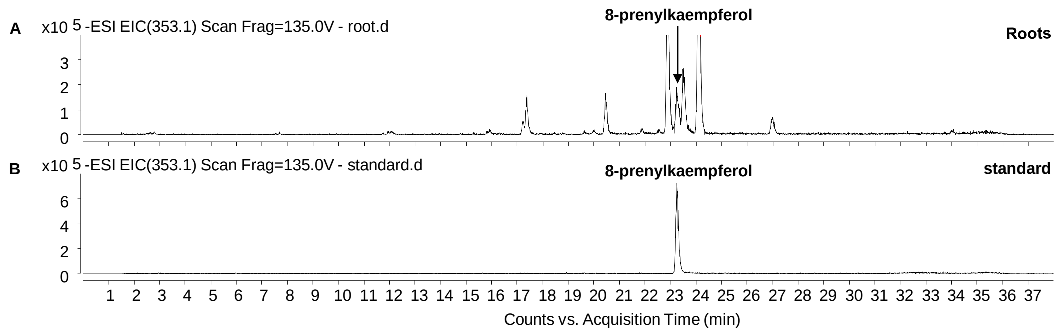

Figure S8. Identification of the 8-prenylkaempferol in roots of white lupin in comparison with the authantic standard by UPLC/MS analysis.

(A-B) Mass spectrum of the roots metabolites by searching the ion fragment with *m/z* of 353.1 under negative ion mode in the roots sample (A) in comparison with the authentic 8-prenylkaempferol standard (B).

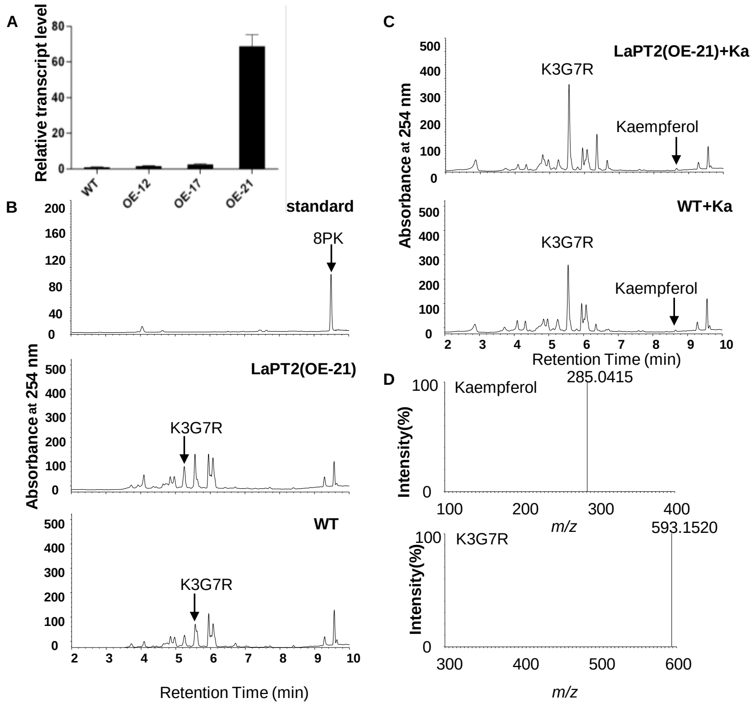

Figure S9. Analyses of the *A. thaliana* plants over-expressing *LaPT2* gene in comparison with the wild type control.

(A) Detection of expression level of *LaPT2* in transgenic *Arabidopsis* by qRT-PCR with triplicates. (B) Representative HPLC chromatographs of the flavonoid profiles in seedlings of the transgenic lines (LaPT2, middle), and the wild type (WT, lower) in comparison with standard 8-prenylkaempferol (upper). (C) Representative HPLC chromatographs of the flavonoid profiles in seedlings of the transgenic lines (LaPT2, upper), and the wild type (WT, lower) feeding with kampferol. (D) Mass spectrum of kaempferol (upper) and K3G7R (lower).
